# Targeting Leukemic Stem Cells in Chronic Myeloid Leukemia: Is It Worth the Effort?

**DOI:** 10.3390/ijms22137093

**Published:** 2021-06-30

**Authors:** Simona Soverini, Sara De Santis, Cecilia Monaldi, Samantha Bruno, Manuela Mancini

**Affiliations:** 1 Dipartimento di Medicina Specialistica, Diagnostica e Sperimentale Università di Bologna, 40138 Bologna, Italy; sara.desantis3@studio.unibo.it (S.D.S.); cecilia.monaldi@studio.unibo.it (C.M.); samantha.bruno2@unibo.it (S.B.); 2 Istituto di Ematologia “Seràgnoli”, IRCCS Azienda Ospedaliero-Universitaria di Bologna, 40138 Bologna, Italy; mancini_manu@yahoo.com

**Keywords:** chronic myeloid leukemia, *BCR-ABL1*, tyrosine kinase inhibitors, stem cells, bone marrow microenvironment, niche, drug resistance

## Abstract

Chronic myeloid leukemia (CML) is a classical example of stem cell cancer since it arises in a multipotent hematopoietic stem cell upon the acquisition of the t(9;22) chromosomal translocation, that converts it into a leukemic stem cell (LSC). The resulting *BCR-ABL1* fusion gene encodes a deregulated tyrosine kinase that is recognized as the disease driver. Therapy with tyrosine kinase inhibitors (TKIs) eliminates progenitor and more differentiated cells but fails to eradicate quiescent LSCs. Thus, although many patients obtain excellent responses and a proportion of them can even attempt treatment discontinuation (treatment free remission [TFR]) after some years of therapy, LSCs persist, and represent a potentially dangerous reservoir feeding relapse and hampering TFR. Over the past two decades, intensive efforts have been devoted to the characterization of CML LSCs and to the dissection of the cell-intrinsic and -extrinsic mechanisms sustaining their persistence, in an attempt to find druggable targets enabling LSC eradication. Here we provide an overview and an update on these mechanisms, focusing in particular on the most recent acquisitions. Moreover, we provide a critical appraisal of the clinical relevance and feasibility of LSC targeting in CML.

## 1. Introduction: Why Aren’t We Happy Yet with Clinical Results in CML?

Chronic myeloid leukemia (CML) is one of the first human malignancies whose molecular pathogenesis could be fully unraveled, between the 1960s and the 1990s, well before the advent of omics [1]. The t(9;22)(q34;q11) chromosomal translocation, that is consistently detectable in the bone marrow cells of virtually all the patients who are diagnosed with CML, gives rise on chromosome 22 derivative (named ‘Philadelphia’ after the city where it was first observed in karyotypes of CML patients) to a fusion gene, *BCR-ABL1*, that encodes a protein with deregulated tyrosine kinase activity. CML is also one of the first human malignancies against whom molecularly targeted therapies could successfully be applied [1]. Once the central role of this gain of function alteration in the initiation and maintenance of CML was fully elucidated, it became clear to researchers that BCR-ABL1 was an ideal target of therapy. Starting from the 90’s, large scale screenings of compounds with tyrosine kinase inhibitory activity were started, from which imatinib mesylate emerged as the most promising one [2]. Since then, three generations of ATP-competitive tyrosine kinase inhibitors (TKIs) have been developed that bind in the cleft between the N-terminal and C-terminal lobe of the kinase and effectively turn off its activity. The development and approval of TKIs opened a new era for both patients and physicians [3]. First, they profoundly modified the natural history of the disease, that used to inexorably progress (unless treated with an allogeneic transplant) from a relatively indolent chronic phase (CP) that may last for years, where proliferation is enhanced but terminal differentiation is maintained, to the blastic phase (BP), that fully resembles an acute leukemia and has a dismal outcome [4]. Nowadays, disease progression may still occur in some patients as a consequence of TKI resistance (that is, lack or loss of response to TKI therapy) [5], but it is much rarer. Second, TKIs revolutionized the outcome of CML, transforming a once fatal malignancy into a sort of chronic condition, with patients who achieve optimal response to therapy having a near-normal life expectancy [6] and a strikingly improved quality of life as compared to the previous gold standard of care, i.e., interferon-alpha. For years, it had been envisioned that TKIs had to be administered chronically to CML patients—like insulin to diabetic patients—in order to keep the disease in check. However, in 2010, after a series of anecdotal reports and experiences, a pioneer clinical study by Mahon et al. [7] provided the proof of concept that TKI therapy may be interrupted after a number of years, once the patient has achieved a stable, very ‘deep’ response (measured at the molecular level in terms of logarithmic reduction in *BCR-ABL1* transcripts in peripheral blood (PB) [8]; see Table 1 for detailed description of response definitions in CML).

Deep Molecular Response (DMR) can be further stratified according to the different depth of response. MMR and DMR hold prognostic significance. Additionally, DMR is considered the gateway to Treatment-Free Remission (TFR).

This was another revolution, since it opened our minds to the possibility that an ‘operational cure’ (a term coined by the late John Goldman to indicate a condition in which the patient still harbors residual CML cells, yet he/she maintains remission) could be obtained. Thus, over the past decade, efforts in CML treatment have concentrated on the achievement of deep molecular response (DMR), not only because it correlates with the best possible outcome, but also because it is considered to be the pre-requisite for successful TKI discontinuation, that is defined as ‘treatment-free remission’ (TFR) [9]. How to improve DMR rates and how to increase the proportion of patients who can enter TFR has been the focus of a massive number of clinical trials over the past decade. TFR is so attractive for both patients and clinicians because it improves quality of life, it enables parenting in younger patients, it avoids long-term toxicities/side effects and last but not least, it is associated with significant savings (now imatinib is generic, but second- and third-generation TKIs are very expensive). TFR has been explored in more than 30 clinical trials so far, that investigated the best therapeutic strategies to induce DMRs and how deep and prolonged DMRs should be [10]. As a consequence, although studies differed in terms of design and inclusion criteria, nevertheless, the results were all strikingly concordant in showing that TFR is at present successful in only 40 to 60% of the patients.

As for many other forms of leukemia, the stem cell (SC) origin of CML is well known. Normal hematopoiesis is sustained throughout one’s life by the regulated proliferative and differentiation activity of a large pool of hematopoietic stem cells (HSCs). Leukemic SCs (LSCs) originate when a transforming mutation (in the case of CML, the *BCR-ABL1* oncogene) is acquired by an HSC. The study of LSCs in CML—together with the study of the bone marrow microenvironment (BMM) that is known to be their sanctuary—has always been one of the most active lines of research. Initially because LSCs were considered to be the reservoir from whom resistance to TKI therapy—a problem that was more urgent before the advent of second- and third-generation TKIs—may arise. More recently, because LSC persistence might be the reason, or at least contribute to molecular recurrence in all those patients who fail to achieve TFR. A number of publications have reviewed the biology of LSCs in CML, summarizing more than 20 years of knowledge. Here, we will provide an update including the most recent acquisitions, in particular those related to druggable targets. Moreover, based on the current clinical and biological evidences, we will discuss the relevance and the feasibility of LSCs and/or BMM targeting, pointing out the controversies and the gaps yet to be filled.

## 2. LSCs: The Ultimate Enemy in CML?

**Figure 1:**
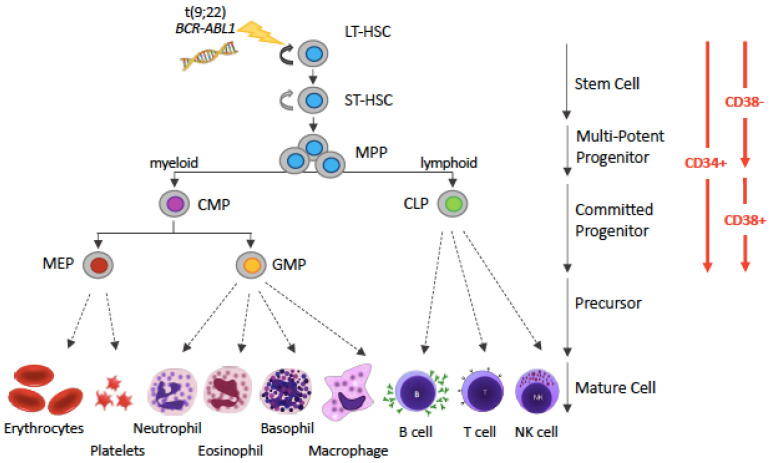
Simplified representation of the hematopoietic hierarchy. Hematopoiesis follows a hierarchical organization with hematopoietic stem cells (HSCs) on top and mature blood cells at the bottom. HSCs are the sources of various types of progenitor cells that proliferate extensively, forming more differentiated cells at the expense of their self-renewal capacity. The various types of precursor cells have been omitted, for simplicity (and this is denoted by dotted arrows instead of solid arrows). The driver lesion of CML, the t(9;22) chromosomal translocation giving rise to the *BCR-ABL1* fusion gene, is gained at the HSC level. Abbreviations: LT-HSC: long-term hematopoietic stem cell; ST-HSC: short-term hematopoietic stem cell; MPP, multi-potent progenitor; CMP, common myeloid progenitor; CLP, common lymphoid progenitor; MEP, megakaryocyte-erythrocyte progenitor; GMP, granulocyte-monocyte progenitor.

The SC origin of CML is well documented. LSCs of CP CML are thought to lie within the haematopoietic stem cell (HSC) population immunophenotypically defined as positive for CD34 and negative for CD38 surface markers (CD34+CD38−) [11] that stands atop the haematopoietic hierarchy (Figure 1).

In contrast, LSCs in BP CML can originate either from HSCs or from cells at later stages (i.e., progenitors) [12]. However, the finding that some putative LSCs lack leukemogenic potential [13] and that individuals who have *BCR-ABL1* transcripts detectable at low levels in their peripheral blood (PB) but are otherwise healthy were observed in some old studies [14,15], suggest that there is heterogeneity in LSCs and that the generation of LSCs from HSCs and their ability to function as leukemia initiating cells (LICs) are the result of an interplay between cell-intrinsic and cell-extrinsic factors. For example, an RNA-seq analysis found that gene expression profiles associated with activated immune response were downregulated in *BCR-ABL1*-positive HSCs from mice that developed leukemia as compared to those from mice that did not [16]—pointing to a role for the immune response in regulating the leukemogenic potential of *BCR-ABL1*-positive LSCs.

The first direct evidence in CML patients of a deeply (but reversibly) quiescent subpopulation of leukemic cells with both in vitro and in vivo SC, properties provided by Tessa Holyoake, dates back to 1999 [17]. It was later found that these cells are resistant to TKI-induced apoptosis, thus are not eliminated by therapy [18] and that normal hematopoiesis is rather restored by TKI treatment through removal of the proliferative advantage of CML progenitors [19]. In line with such evidences, BCR-ABL1-positive LSCs were detected in patients in long-term remission after imatinib treatment, since *BCR-ABL1*-positive CD34+CD38– cells isolated from such patients were found to have long-term repopulating capacity in immunocompromised mice [20,21,22,23]. It was later found that these cells are not killed by TKIs since they do not depend upon BCR-ABL1 kinase activity for their survival [24,25]. In these cells, BCR-ABL1 is turned off or is expressed at very low levels [26,27].

The inherent CML LSC insensitivity to TKIs led to the assumption that disease eradication, desirable to avoid unpredictable relapses and to enhance the possibility of TFR, could be achievable if a comprehensive knowledge of cell-intrinsic and cell-extrinsic mechanisms of LSC survival was obtained. This fostered intensive investigations of BCR-ABL1 kinase-independent pathways that could sustain LSC survival. In parallel, a number of studies focused on the CML BMM. The interdependency between LSCs and the BMM where they reside (niche), the fact that BMM profoundly modifies the fitness and the sensitivity to (chemo)therapeutic agents of LSCs and that LSCs, in turn, remodel the BMM were indeed well known [28]. Thus, in an attempt to devise novel weapons to kill CML LSCs, over the past two decades hundreds of studies have been conducted, that have contributed to the accumulation of a significant wealth of information. Similarly, the putative roles played by the different cell types that inhabit the BMM (osteoblasts, osteoclasts, mesenchymal cells, endothelial cells, etc.) and their cross-talk with LSCs via direct contact interactions as well as via secretion of soluble factors or extracellular vesicles have been intensively explored. In the following sections, we will review current knowledge, with a dedicated focus on the latest acquisitions about LSCs and BMM in CML that contributed to add further pieces to an apparently never-ending puzzle.

## 3. Aberrant Surface Markers in CML LSCs and Their Diagnostic, Predictive and Therapeutic Role

The CD34+CD38− immunophenotype is shared by CML LSCs and by normal HSCs. It has been demonstrated that residual normal long-term clonogenic CD34+CD38− are detectable in CML patients at diagnosis, and that the proportion of LSCs varies greatly across different patients. Thus, discrimination between leukemic and normal long-term clonogenic CD34+CD38− cells is needed. A multiparameter flow cytometry (MPFC) study showed that normal (*BCR-ABL1*-negative) long-term colony-forming cells in CML patients were CD34/CD45^low^, had low light scatter properties, lacked lineage marker expression and were CD90^dim^ [29]. Remarkably, both their light scatter as well as their CD90 expression were lower than that of CD34+38− cells in normal BM. In contrast, leukemic long-term colony forming cells were CD34/CD45^high^, had higher forward and sideward scatter, variable lineage marker and higher CD90 expression. The presence of detectable residual normal stem cells identified with such gating strategies at diagnosis correlated with lower grade 3 and 4 hematological toxicity and with optimal response to imatinib [29]. The potential predictive value of LSC assessment was further showed by the Nordic group [30]. In a pilot phase 2 study in 46 CML patients, Mustjoki et al. [31] used cell sorting followed by fluorescence in situ hybridization (FISH) to evaluate the LSC fraction in terms of BM cells with a CD34+CD38− and Philadelphia-positive phenotype at diagnosis and at +1, +3 and +6 months from start of treatment with imatinib or dasatinib. They found that all patients who did not achieve MMR at 18 months (that is defined as an optimal response according to international guidelines [32]) had >75% of Philadelphia-positive cells in the CD34+CD38− fraction at diagnosis. Moreover, both patients who progressed during the study period had more than 90% of Ph+ cells in the CD34+CD38− compartment. The authors concluded suggesting that LSC burden might serve as a novel prognostic biomarker and may prove useful in identifying CML patients who would benefit from treatment intensification. In a subsequent study [33], MPFC and cell sorting followed by FISH were used in parallel to evaluate the LSC burden in a subset of 50 CML patients enrolled in the international Evaluating Nilotinib Efficacy and Safety as First-Line Treatment (ENEST-1st) clinical trial. The primary objective of the study intended to answer the question whether the composition of the CD34+CD38− compartment at diagnosis and during follow-up could be predictive of MR^4^ achievement at 18 months. The findings confirmed that, whatever the method, the quantitation of LSCs in patients at diagnosis reflects the ‘aggressiveness’ of the disease (in terms of blast count, spleen size, Sokal score, etc.), correlates with therapy-related hematological toxicity, and most importantly, allows to predict the achievement of key response milestones during therapy with nilotinib.

Over the past decade, additional, aberrant surface markers have been identified and validated that better discriminate between residual normal HSCs and CML LSCs. Needless to say that finding highly specific, robust and reproducible markers would hold an immense application potential, since this could improve diagnosis and facilitate isolation and biomolecular/functional characterization of LSCs. Additionally, beyond the already discussed prediction of response, it can be hypothesized that accurate estimation of LSCs may empower residual LSC tracking and quantitation during and after therapy, also as a predictor of TFR success. Last but not least, finding specific targets expressed on the surface of CML LSCs but not on normal HSCs or other cell types would enable highly selective therapeutic targeting of LSCs [34]. A variety of immunotherapy strategies, like cell-targeting antibodies, antibody-toxin conjugates, bispecific antibodies, and chimeric antigen receptor-engineered T (CAR-T) cell-based strategies may indeed be evaluated in an attempt to eradicate the disease, and these are likely to have fewer off target or toxic effects than inhibitors of pathway or players essential for LSC survival but often playing key roles also in normal cells.

In recent years, immunophenotypic studies followed by functional validation have proposed a series of novel surface markers that, alone or in combination, might prove extremely useful to refine the identification and the enumeration of CML LSCs within the CD34+CD38− fraction, and in some cases, even enable their therapeutic targeting. The first such marker to be identified is IL1RAP. IL1RAP serves as co-receptor of the interleukin 1 receptor (IL1R1), but its exact functions remain unknown. Almost all CD34+CD38− BCR-ABL1-positive cells express IL1RAP, while BCR-ABL1-negative cells lack IL1RAP expression of their surface [35]. Recently, it has been reported that estimation of the LSC burden at diagnosis by quantitation of the percentage of IL1RAP-positive cells within the CD34+CD38− compartment may predict TKI response in terms of complete cytogenetic response (CCyR; the disappearance of the Philadelphia chromosome in bone marrow metaphases; Table 1) and major molecular response (MMR; defined as 3-log reduction in *BCR-ABL1* transcript levels from the standardized baseline, as assessed by real time quantitative polymerase chain reaction; Table 1) [36]. Moreover, it has been shown that CAR-T cells directed against IL1RAP can successfully be generated and are effective in vitro and in a xenograft mouse model against both LSCs and, to a lesser extent, monocytes expressing IL1RAP, with no apparent effect on the hematopoietic system, including CD34+ stem cells [37]. This study thus provided a proof of concept that IL1RAP is a tumor-associated antigen that can be exploited for a CAR T-cell-based immunotherapy approach in CML. Such an approach might be envisioned to be further explored in primary TKI-resistant or -intolerant patients and in patients who are candidate to allograft.

Later, the same group performed an RNA-seq study aimed to identify additional, cell surface markers differentially expressed between CML CD34+CD38-, CD34+CD38+ and their counterparts in normal BM that identified CD36 as specifically upregulated in primitive CML cells [38]. They observed that CD36 expression could separate CD34+CD38-IL1RAP+ cells into two distinct subpopulations, with the CD36+ subpopulation being more quiescent and insensitive to imatinib that the CD36- one. Finally, they found that CD36-targeting antibodies were effective in killing CD36-expressing KU812 cells by antibody dependent cellular cytotoxicity.

Another very promising marker is CD26, or dipeptidylpeptidase-IV. CD26 is a cell surface enzyme that is responsible for the proteolytic degradation of various cytokines including IL-3, GM-CSF and SDF-1 [39]. The group of Peter Valent first reported that nearly all CD26+ LSCs express BCR-ABL1, whereas the CD26─ SC fraction from the same patients is negative for BCR-ABL1 [40]. CD26 is also expressed on LSCs of myeloid and lymphoid BP and on the Philadelphia chromosome-positive acute lymphoblastic leukemia subtype harboring p210*^BCR-ABL1^* [41]. Moreover, Herrmann et al. showed that CD26 is not detectable on normal BM SCs, nor is it expressed on LSCs in other hematopoietic malignancies [40]. CD26+ cells isolated from CML patients exhibited long-term proliferation activity in vitro and repopulation activity in immunocompromised mice—the classical tests used to define bona fide LSCs. Thus, CD26 can be considered as a highly specific marker for CML LSCs, and this holds true for all stages of the disease. In two studies, decrease in CD26+ cells was found to correlate with response to TKI therapy [40,42]. Based on these findings, Bocchia et al. hypothesized that noninvasive cytofluorimetric measurement of CD26 in PB might be used to explore the persistence of circulating LSCs in CML patients [43]—which may prove useful to study the impact of residual LSCs on the possibility to successfully achieve TFR. In a first, large cross-sectional study of more than 400 CML patients at diagnosis, on TKI treatment or in TFR [44], they found that CD26 expression could be detected by multiparametric flow cytometry analysis of PB CD45+/CD34+/CD38− cells not only (as expected) at diagnosis, but also in patients with DMR, as well as in a significant proportion (66%) of patients in TFR. The fact that the absolute number of circulating CD26+ cells did not mirror BCR-ABL1 transcript levels as assessed by real time PCR of PB leukocytes, suggested that, in CML, minimal residual disease (MRD) assessment seems not to provide a reliable estimate of the actual residual LSC burden. Moreover, the fact that the majority of patients in TFR had detectable circulating LSCs appears to support the hypothesis that cell-extrinsic factors contribute to avoid disease recurrence. In a second ongoing prospective study, whose interim results on 176 patients have been presented at the last (2020) meeting of the American Society of Hematology, Bocchia et al. [45] confirmed the absence of a correlation between the absolute number of CD26+ cells/mL and the depth of response in terms of reduction in BCR-ABL1 transcript level. However, they observed that at diagnosis, high CD26+ LSCs number correlated with subsequent failure or suboptimal response to therapy. Moreover, hypothesizing a role of the immune system in the control of residual LSCs, they also assessed PD-L1 expression on CD26+ LSCs and found that, at diagnosis, high PD-L1 expression correlated with reduced probability to achieve an optimal response—in line with the role of PD-L1 in suppressing anti-leukemic T cell response.

The potential role of CD26 as therapeutic target has also been explored. Indeed, it was noted that CD26 is already a therapeutic target in type 2 diabetes mellitus, and that a class of drugs (the gliptins) had already been developed and were routinely used in the clinic. However, a preclinical study evaluating the combination of TKIs (imatinib or nilotinib) with vildagliptin failed to evidence major cooperative effects on engraftment in mice models [46]. More recently, Houshmand et al. [47] developed an innovative strategy based on a liposome loaded with the BCL2 inhibitor venetoclax exploiting begelomab (an anti-CD26 antibody) to selectively target CD26+ CML LSCs. BCL2 is overexpressed and hyperactivated in CML LSCs [48] and BCL2 targeting was indeed found to reduce LSC engraftment in mice [49]. Houshmand et al. [47] found that their immunoliposome could indeed reduce cell growth and induce apoptosis in CD26+ cell lines, and co-administration with TKIs (imatinib, nilotinib) demonstrated synergistic effects. Moreover, they observed that their immunoliposome could dramatically reduce the percentage of CD26+ cells in primary patient samples.

Another recently identified marker is the interleukin-2 (IL-2) receptor alpha chain CD25 [50,51]. The IL-2 receptor on lymphohematopoietic cells is a multimeric complex consisting of an alpha chain (CD25), a beta chain (CD122) and a gamma chain (CD132). CML LSCs are positive for CD25, but do not express substantial amounts of either CD122 or CD132, and, accordingly, are not responsive to IL-2 [52]. Since CD25 is also expressed by AML LSCs, and even normal BM SCs may occasionally display CD25, additional diagnostic LSC markers, such as CD26 and IL1RAP, should be used in combination. Expression of CD25 on CML LSCs seems to result from BCR-ABL1-dependent STAT5 activation, and inhibition of BCR-ABL1 with nilotinib or ponatinib (but not with imatinib) was found to downregulated CD25 expression on LSCs [51]. However, although BCR-ABL1-activated STAT5 is a feature of all clonal CML cells, CD25 is only upregulated and expressed aberrantly on LSCs, while more mature cells like progenitors (CD34+CD38+) display very low if no CD25 positivity. From a functional point of view, CD25 was found to suppress the growth of stem and progenitor cells in CML [51]. Thus, it was hypothesized that LSC insensitivity to TKIs might be facilitated by drug-induced downregulation of CD25, and this sparked searches for drugs capable to induce CD25 re-expression. The dual phosphoinositide-3-kinase (PI3K) and mammalian target of rapamycin (mTOR) blocker BEZ235 and the mTOR inhibitor rapamycin were found to be two such drugs [51].

**Figure 2:**
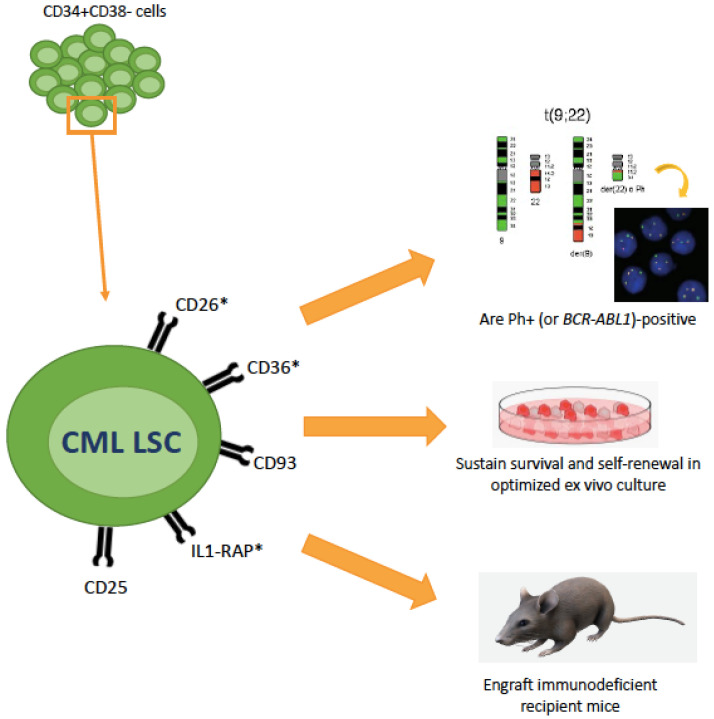
Recent advances in the characterization of CML LSCs markers (and therapeutic targets). CML LSCs lie in the CD34+CD38– cellular fraction but represent only a minority of these cells. Additional surface markers that have recently been identified are shown here. The asterisk denotes whether these markers have also been explored as therapeutic targets. These markers have been shown useful to isolate a population that is BCR-ABL1-positive and functionally behave as LSCs.

Other surface markers expressed more or less specifically on CML LSCs, but not or only at a low level on normal BM stem cells, include CD56 and CD93 [53,54]. Recently, a study by Kinstrie et al. [55] has shown that CD93 is consistently and selectively expressed on a CD34+CD38−CD90+ subpopulation that engrafts patient-derived xenograft models (in contrast to the CD93− counterpart, that fails to engraft) and displays a SC-like signature of gene expression. CD93-expressing cells were not eliminated by TKIs and persisted in patients even after prolonged treatment. Comparison between patients in TFR who did or did not experience molecular recurrence showed that the former harbored detectable CD93+ cells within the CD34+CD38−CD90+ fraction whereas the latter did not. Given that CD93 does not only mark CML LSCs but can also be found on platelets, on endothelial cells and on other cell types, it is highly unlikely that CD93 may serve as a therapeutic target. However, further studies aimed to investigate whether CD93 may be used as predictive biomarker to distinguish those CML patients at high risk of molecular recurrence after discontinuation are warranted. Surface markers that enable to isolate CML LSCs within the CD34+CD38− are summarized in Figure 2.

## 4. Cell-Intrinsic Survival Pathways

As elegantly demonstrated by Corbin et al. [24] and by Hamilton et al. [25], CML LSCs are not BCR-ABL1-addicted for their persistence. This finding reinvigorated the efforts aimed to understand CML LSC biology, in an attempt to identify BCR-ABL1-kinase independent pathways that could be targeted. A number of LSC-specific pathways and players have indeed been identified. Their role in CML LSCs has already extensively been reviewed [32]. A quick overview follows, integrating consolidated concepts with the most recent acquisitions. For clarity, these pathways are listed individually, but it is important to bear in mind that most of them are closely interconnected and interdependent, and converge on a small set of key effectors—mainly transcription factors like Signal Transducer and Activator of Transcription 3/5 (STAT3/5), Forkhead transcription factors of the class O 3A (FOXO3A), β-catenin, p53, c-MYC, NOTCH, BMI-1 and others, that are master regulators of apoptosis, self-renewal, cell fate, senescence.

- Sonic Hedgehog (Hh) pathway: Hh is a master regulator of self-renewal of normal SCs as well as LSCs. The binding of Hh ligand to the Patched (PTCH) receptor relieves the inhibition of the G protein–coupled receptor Smoothened (SMO) that, in turn, activates the transcription factor GLI1. GLI1 then translocates into the nucleus, where it modulates the transcription of several target genes implicated in cell cycle regulation and apoptosis (*CCND1*, *MYC*, etc.) and promote MDM2-dependent degradation of p53. The pathway is hyperactivated in CML LSCs through the upregulation of SMO [56,57].
- Wnt/β-catenin: nuclear β-catenin, a co-activator of the T-cell factor/lymphoid enhancer-binding factor (Tcf/Lef) transcription factors, is required for self-renewal and survival of normal HSCs through the activation of key genes like NOTCH [58] and is, similarly, a key mediator of LSC survival [59]. Both canonical and noncanonical Wnt pathways result in relocation of β-catenin in the cytoplasm, where it is ultimately phosphorylated by Glycogen Sinthase Kinase 3β (GSK3β) and targeted for degradation by an Axin-mediated multimeric complex. Other pathways, like CD70/CD27 [60,61] have been shown to converge on Wnt, thus ultimately influencing β-catenin levels and activity. Noncanonical Wnt pathways, that convey signals in a β-catenin-independent fashion, have also been implicated in LSC survival. Via activation of phospholipase C (PLC), Wnt has indeed been shown to increase intracellular Ca2+, leading to the activation of the Ca2+-calmodulin-dependent protein phosphatase calcineurin and ultimately resulting in the activation of Nuclear Factor of Activated T-cells (NFAT) transcription factor [62]. Enhanced NFAT activity mediated by this noncanonical Wnt/Ca2+ signaling increases autocrine cytokine production that provides to LSCs compensatory prosurvival signals upon exposure to TKIs.
- PI3K/AKT: the PI3K/AKT pathway mediates proliferation and is physiologically activated by binding of growth factors to receptor tyrosine kinases. BCR-ABL1-dependent activation of PI3K/AKT signaling contributes to CML LSCs maintenance, as it leads to AKT-mediated phosphorylation and cytosolic retention of FOXO, hence blocks the transcription of FOXO target genes involved in apoptosis [63]. Among others, transforming growth factor β (TGF-β) signaling influences FOXO nuclear translocation via PI3K/Akt signaling [63].
- Janus kinases (JAK): in CML, JAK2 signaling has been shown to converge on STAT3 and STAT5 activation. STAT5 phosphorylation and translocation into the nucleus is well known to play a key role in CML pathogenesis [64,65]. STAT3 signaling has also been implicated in CML LSC survival [66,67,68]. Both JAK2 and JAK1 have been implicated in STAT3 activation.
- Protein phosphatase 2A (PP2A): the tumor suppressor *PP2A* gene encodes a multimeric serine/threonine phosphatase that is inactivated via overexpression of its endogenous inhibitors SET and CIP2A. In CML, PP2A loss as a result of BCR-ABL1-dependent expression of SET has been implicated in quiescent LSC maintenance [64]. BCR-ABL1 expression (but not activity) was found to be essential for the recruitment of JAK2, activation of a JAK2/β-catenin survival/self-renewal pathway.
- Promyelocytic leukemia (PML): the *PML* gene, involved in the t(15;17) chromosomal translocation of acute promyelocytic leukaemia, encodes a protein localizing to nuclear bodies that acts as a tumor suppressor, controlling apoptosis, cellular proliferation and senescence [69] and negatively regulating mTOR [70]. PML has been shown to play an indispensable role in maintaining LSC quiescence in CML [71].
- Dual Specificity Tyrosine Phosphorylation Regulated Kinase 2 (DYRK2): the KLF4 transcription factor has been shown to sustain CML LSCs via downregulation of the dual-specificity kinase DYRK2, that mediates the stabilization of p53 and the proteasomal degradation of c-MYC. This results in reduced apoptosis and increased self-renewal [72]. The central role of p53 and c-MYC in LSC maintenance had already been elegantly demonstrated by a seminal proteomic study conducted by Tessa Holyoake’s group [73].
- Micro RNAs (miRNAs): comparative miRNA expression profiling has highlighted several deregulated miRNAs in CML LSCs. For example, the BCR-ABL1 kinase-independent upregulation of miR-29a-3p and miR-660-5p was observed in the CD34+CD38- fraction, and via the downregulation of their respective targets Ten Eleven Translocation 2 (TET2) and Endothelial PAS Domain Protein 1 (EPAS1) was found to confer TKI resistance in vitro [74]. More recently, Pellicano et al. [75] have shown that BCR-ABL1 kinase-dependent upregulation of hsa-mir183 results in the downregulation of its direct target Early Growth Response 1 (EGR1) transcription factor, and, as a consequence, upregulation of the cell cycle regulator E2F1, that is known to control both cell proliferation and p53-dependent/independent apoptosis. The latter was found to play a pivotal role in controlling LSC (but not normal HSC) survival and proliferation, since E2F1 inhibition led to a decrease in colony-forming potential, cell-cycle arrest, and induction of p53-mediated apoptosis. Even more recently, it has been reported that miR-196a-5p is also upregulated in the CD26+CD34+CD38– fraction as compared to the CD26–CD34+CD38–, although the roles of this microRNA in CML LSCs have yet to be elucidated [76]. BCR-ABL1-independent signaling pathways have also been shown to impact on the biogenesis and/or on the levels of miRNAs implicated in LSC survival and resistance. JAK/STAT-dependent activation of Adenosine Deaminase Acting on RNA1 (ADAR1), implicated in post-transcriptional adenosine-to-inosine RNA editing, has been shown to impair the biogenesis of let-7, ultimately enhancing LSC self-renewal [77]. Additionally, miR-21, that appears to be under the control of the PI3K/AKT pathway, has been implicated in LSC resistance to TKIs [78]. The role of miR-30a in autophagy will be discussed below. Further investigations into the roles of miRNAs as well as of other noncoding RNAs in LSC intrinsic survival mechanisms are warranted.
- Autophagy: it is an evolutionarily conserved catabolic process that physiologically consists in the lysosomal degradation and recycle of unnecessary cellular components to generate adenosine triphosphate (ATP) and essential building blocks during nutrient and/or oxygen deprivation. Autophagy has been implicated in normal HSC maintenance [79], but may also act as a pro-survival pathway that helps tumor cells tolerate metabolic stress and avoid apoptosis induced by anticancer agents [80]. Similarly, in CML, autophagy has been highlighted as a drug resistance pathway employed by LSCs for their survival [81]. BCR-ABL1 represses autophagy, in part via the PI3K/Akt/mTORC1 pathway and in part via induction of miR-30a, that in turn dowregulates two key autophagy genes, *Beclin 1* and *ATG5*. TKI treatment induces autophagy, and inhibition of autophagy results in enhanced TKI-induced killing of stem and progenitor cells [81].
- Epigenetic alterations: Enhancer of Zeste Homolog 2 (EZH2) is a member of the Polycomb Repressive Complex 2 (PRC2) that trimethylates histone H3 at lysine 27 but may also directly regulate gene expression. It has been shown to be highly expressed in CML LSCs and to contribute to their survival [82]. Targeting histone deacetylases was also explored against CML LSCs, in light of the reported efficacy of such a strategy in inducing apoptosis in non-proliferating cells, and gave promising results when tested in combination with TKIs in cellular and mouse models [83]. Sirtuin 1 (SIRT1) deacetylase is a multifunctional protein that has several genes implicated in many cellular pathways, including energy metabolism and stress response, among its targets—including *TP53* and *FOXOs*. SIRT1 has been shown to be overexpressed in CML LSCs and to contribute to LSC resistance to TKIs [84,85]. Later on, SIRT1 was found to mediate increased mitochondrial oxidative phosphorylation in CML [86], an important survival mechanism of LSCs [87]—see Section 5. The histone acetyltransferase CBP has also been implicated in LSC self-renewal, as opposed to p300 which rather promotes differentiation and senescence. Both can be recruited by b-catenin upon activation of the Wnt pathway. Inhibition of CBP was found to favor the formation of b-catenin/p300 complexes, switching the balance from cell proliferation to differentiation [88].
- Metabolic changes: one of the most recent and intriguing chapters of LSC survival adaptations is centered on metabolism. Besides the well-known role played by the hypoxic conditions of BMM in metabolic rewiring (that will be discussed more in detail in the dedicated section below), several molecules implicated in cellular metabolism have been shown to be deregulated in CML LSCs. Arachidonate 5-lipoxygenase (ALOX5), involved in fatty acid metabolism (it converts arachidonic acid into leukotrienes), has been shown to be required for CML LSC self-renewal [89]. Later on, ALOX15 was also found to be essential for LSC survival [90].
- Autocrine factors: BCR-ABL1-induced transformation is known to rely, among other mechanisms, on the autocrine production by LSCs of cytokines like Interleukin (IL)-3 and Granulocyte-Colony Stimulating Factor (G-CSF) resulting in growth factor-independent STAT5 activation. Other, kinase-independent autocrine loops have more recently been reported to contribute to LSC persistence. BCR-ABL1 kinase-independent autocrine production of Tumor Necrosis Factor a (TNFa) has been reported to support CML LSC survival via the nuclear factor κB (NFκB)/p65 pathway leading to the expression of the IL-3 and granulocyte/macrophage-colony stimulating factor common β-chain receptor [91]. An autocrine loop enhancing bone morphogenetic protein (BMP) 2 and 4 (normally produced by mesenchymal cells and involved in regulation of proliferation and fate of normal HSCs) concentration in the BMM (see further details in the next section) has also been implicated in LSC resistance to TKIs [92]. Very recently, CML LSC have also been found to upregulate pleiotrophin (PTN), a heparin-binding growth factor normally produced by BM stromal cells and ECs. PTN has been shown to promote CML LSC survival and TKI resistance [93].

Since in most cases the molecules or pathways listed above were found to be druggable, the same studies showed, in vitro and often in mouse models, very promising efficacy of targeted agents directed against them in sensitizing LSCs to TKI-induced killing or eliminating LSCs via synthetic lethality. This has recently been reviewed in detail by Muselli et al. [94]. Some promising molecules or strategies have also moved forward to clinical trials. A series of trials have investigated, or are investigating, the use of the JAK2 inhibitor ruxolitinib, for whom repurposing would be quite seamless (ruxolitinib is already approved for Philadelphia chromosome-negative myeloproliferative neoplasms), in combination with TKIs in patients with molecular persistence. The combination has proven safe and well tolerated but contrasting results have been reported as far as efficacy is concerned. Whereas a phase 1 study of ruxolitinib in combination with nilotinib conducted at the H. Lee Moffitt Cancer Center and Research Institute has reported encouraging results in terms of MR improvement [95] (and a phase 2 study is now ongoing in patients who have failed a first discontinuation attempt), another phase 1–2 study of ruxolitinib in combination with TKIs has prematurely been terminated by the MD Anderson Cancer Center for lack of efficacy. Two SMO inhibitors, LDE225 (sonidegib) [96] and BMS-833923, had already been tested in clinical trials a decade ago, demonstrating unfavorable safety profiles and a substantial lack of efficacy. Similarly, mTOR and histone deacetylase inhibitors yielded disappointing results in clinical trials. Autophagy inhibitors were also among the first compounds to be evaluated in the clinic. The CHOICES (CHlOroquine and Imatinib Combination to Eliminate Stem cells) study was activated as early as in 2009, and results have just recently been published [97]. It was an international randomized phase II trial aimed to evaluate the safety and efficacy of a combination of imatinib and hydroxychloroquine (an antimalarial drug that was found to be an autophagy inhibitor) versus imatinib alone for CML patients with detectable residual disease. Although the combination was relatively well tolerated, the improvement in molecular response it brought was modest. Moreover, a high risk of retinopathy is known to be correlated to long-term exposure to the drug. Therefore, more potent and selective autophagy inhibitors, such as Lys05 [98], have been proposed. PRI-724 (also known as ICG-001), that antagonizes Wnt/β-catenin/TCF-mediated transcription and specifically binds to CREB-binding protein (CBP) [99] has been evaluated in a phase 2 study in patients with advanced myeloid malignancies, including CP CML patients with failure of 2 TKIs (where PRI-724 has been administered in combination with dasatinib). The study has recently been terminated, but results have not yet been published. More recently, pioglitazone, an antidiabetic drug that is a Peroxisome Proliferator-Activated Receptor gamma (PPAR-g) agonist and targets quiescent LSCs in vitro by decreasing transcription of STAT5, has been tested in addition to imatinib in patients failing to achieve MR^4.5^ on imatinib alone [100]. Results have been encouraging, so that a new study where the combination is evaluated in preparation of a second TFR attempt in patients who failed a first one is ongoing (PIO2STOP). On the other hand, a study where pioglitazone was tested in association with imatinib to improve TFR rates failed to meet its endpoint [101].

It is also believed that potentiating the immunological recognition and elimination of CML LSCs may represent an easier, effective therapeutic approach aimed to LSC eradication. The insensitivity of CML cells to apoptosis is not absolute since it can be overcome by immune-mediated mechanisms, as demonstrated by the graft versus leukemia effect provided by the allogeneic transplantation. It has been demonstrated that T-lymphocyte-mediated killing of target cells via Fas-receptor (Fas-R) triggering plays an important role in the elimination of malignant cells, including CML cells [102]. CD34+ CML cells have been shown to express Fas-receptor (FAS-R) or CD95, and interferon-α (IFN-α) therapy has been found to further upregulate FAS-R expression. FAS-R agonists were also found to inhibit colony formation in CD34+ progenitors [102].

**Figure 3:**
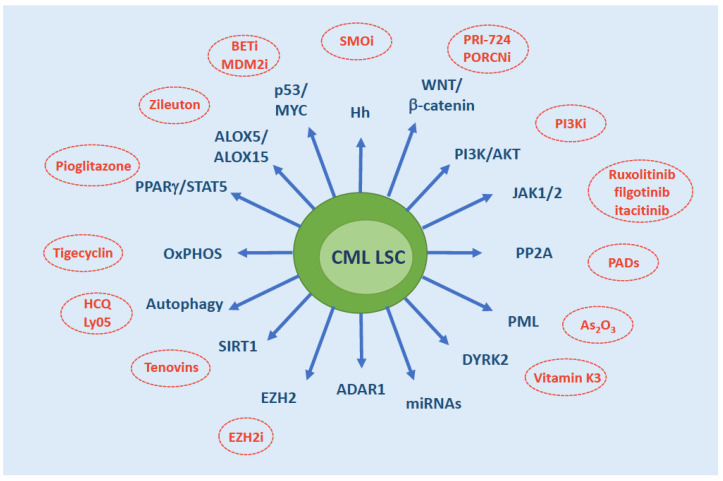
Overview of the molecules/pathways that have been implicated in CML LSC persistence. Therapeutic strategies that have demonstrated efficacy in vitro or in mouse models are indicated. Abbreviations: SMOi, SMO inhibitors; PORCNi, Porcupine inhibitors; PI3Ki, PI3K inhibitors; PADs, PP2A activating drugs; EZH2i, EZH2 inhibitors; HCQ, hydroxychloroquine; BETi, BET inhibitors; MDM2i, MDM2 inhibitors; OxPHOS, oxidative phosphorylation.

A variety of phytochemicals have been shown to act synergistically with conventional anti-cancer drugs in the elimination of cancer SCs. Isoflavones, polyphenols, quercetins etc have been reported to interfere with and effectively target WNT, b-catenin, FOXO, NFkB, MAPK, autophagy and other key players in LSC survival (reviewed in [103,104]). The first example of efficacy of a natural compound for the treatment of CML is homoharringtonine (HHT), a naturally-occurring ester of the alkaloid cephalotaxine isolated from various trees of the Cephalotaxus genus [105]. Triptolide [106], berbamine [107], resveratrol [108], the synthetic retinoid derivative fenretinide [109] and others have also been shown to be active in vitro against CML LSCs. Several natural compounds have shown in vitro activity in TKI-resistant CML cell lines, thus it is likely that further future studies will explore their potential against LSCs, too. Concomitant use of phytochemicals and TKIs would hold the advantage of minimizing the side effects normally arising from combination therapy. A summary of the key cell-intrinsic factors sustaining CML LSC survival and their ‘druggability’ is shown in Figure 3.

## 5. Cell-Extrinsic Survival Pathways

As anticipated, the BMM is thought to play a key influence on LSCs, and this is a general piece of knowledge that holds true not only in CML. The cross-talk between LSCs and BMM is mediated by (i) soluble or exosome-incapsulated factors and (ii) cell-cell direct interactions via surface molecules, and involves a broad array of cell types on the BM side, like mesenchymal stromal, endothelial, perivascular, osteolineage and immune cells. LSCs are known to coopt or hijack BM niches, that protect and nurture them at the expense of normal hematopoiesis. In the light of this complex interplay, it is believed that the study of the BM niches is at least as important as the study of LSCs, as a deep understanding of the role of BMM may provide important clues on how to selectively eliminate LSCs.

It is important to consider that the low oxygen tension of BM niches profoundly influences LSC features, first of all their metabolism. Quiescent and self-renewing HSCs rely on glycolysis for energy production. This is an adaptation to the hypoxic environment of the BM niches and reflects the low energetic demands of HSCs. Upon differentiation and lineage commitment, a metabolic rewiring to oxidative phosphorylation (OxPHOS) occurs [110,111]. Cancer SCs have been found to be variably dependent on OxPHOS or on glycolysis, depending on cancer type, for growth and maintenance. In CML LSCs, OxPHOS has been found to be crucial for production of energy and anabolic precursors [87], thus providing an attractive metabolic vulnerability that might be exploited for their selective targeting. Moreover, hypoxia stabilizes Hypoxia-Inducible Factors 1 (HIF1), a set of transcription factors playing a key role in regulating proliferation, maintenance, and survival of LSCs [112].

An altered level of pro-inflammatory cytokines (including IL-1α, IL-1b, IL-6, macrophage inflammatory protein-1 a (MIP-1a), MIP-1b and TNFα) or chemokines has been reported in CML BM niches, and this was found not to be fully corrected upon TKI-induced remission [113,114]. This differentially affects CML LSC and normal HSC in terms of maintenance and growth, providing a competitive advantage to LSCs. For example, levels of C-X-C motif chemokine ligand 12 (CXCL12), implicated in HSC maintenance, have been shown to be reduced in CML BMM [115]. This is detrimental to HSCs but has been found to promote LSC expansion by increasing their self-renewing divisions. As anticipated in the above section, BMP2 and BMP4 are abnormally abundant in CML BMM [116]. Together with overexpression of their receptor BMPR1B, this leads to a strong upregulation of the BMP pathway in CML LSCs that has been implicated in their survival and expansion. Very recently, a high throughput screen of the effects of 313 human cytokines on the growth of CD34+CD38– cells isolated from CML patients has confirmed the previously reported regulatory effects of IL-3, IL-1a/b, GM-CSF and IFNg but has also identified myostatin propeptide (MSTNpp; produced by muscle cells and secreted into the bloodstream where it binds and regulates the myokine myostatin) as a novel positive regulator [117]. It was found that MSTNpp can indeed be detected in the plasma of CML patients and that it may be produced by mesenchymal stromal cells. By binding to a yet unknown receptor on the surface of CML cells, MSTNapp was confirmed to expand primitive cells via STAT5 and SMAD2/3. Besides cytokines and chemokines, other soluble factors have been shown to influence the fitness of LSCs. For example, mesenchymal stromal cells provide exogenous Wnt to activate the Wnt/b-catenin pathway in LSCs [118]. More recently, an intriguing role for several miRNAs secreted in the BMM has been elucidated. Endothelial cells have been found to supply miR-126 to CML LSCs to support quiescence and self-renewal [119]. Indeed, in CML LSCs, miR-126 is downregulated due to the BCR-ABL1-mediated phosphorylation of Sprouty-related EVH1-domain-containing 1 (SPRED1), which leads to inhibition of the RAN-exportin-5-RCC1 complex that mediates miRNA maturation. Thus, TKI-mediated inhibition of BCR-ABL1 results in even greater miR-126 levels, that further strengthen LSC quiescence and persistence. Moreover, expression of the tumor suppressor miR-300, induced by hypoxia and exosomes derived from mesenchymal stromal cells, has recently been found to have dose-dependent, dual antiproliferative and PP2A-activating functions that are essential for induction and maintenance of LSC quiescence [120]. MiR-300-induced loss of cyclin D2/cyclin-dependent kinase 6 (CDK6), which occurs at low levels of *MIR300* expression, likely represents the mechanism by which miR-300 antiproliferative activity contributes to CML stemness.

Direct cell-cell contact interactions between CML LSCs and the BMM have also been extensively investigated. LSC adhesion indeed contributes to their homing and engraftment. An array of interactions involving integrin and selectins, like very late antigen (VLA) and vascular cell adhesion molecule (VCAM), L- and E-selectins and their ligands, have been described [121]. For example, increased expression of CD44 on LSCs and increased binding to E-selectin on BM endothelial cells has been shown to contribute to LSC dormancy and TKI resistance [122]. Recently, use of an E-selectin inhibitor interfering with CD44/E-selectin binding in mice was found to result in an upregulation of SCL/TAL1 transcription factor and in a downregulation of CD44 [123]. Thus SCL/TAL1 was found to be a negative regulator of CD44. CD44 expression was found to be BCR-ABL1-dependent. The overexpression of the NOTCH ligand JAGGED-1 on the surface of osteoblasts has also been shown to promote LSC quiescence [124]. Accordingly, after osteoblast ablation, the quiescent HSC subset is lost, and bone marrow is converted into a proliferation-promoting microenvironment for both normal and malignant SCs.

The study of the BMM in CML stems from the rationale that the same soluble factors, molecules and mechanisms that mediate leukemia–BMM interactions might represent vulnerabilities and might be exploited as potential therapeutic targets. Indeed, several approaches have been evaluated to interfere with LSC homing and engraftment, and many of them have shown synergistic effects with TKIs.

**Figure 4:**
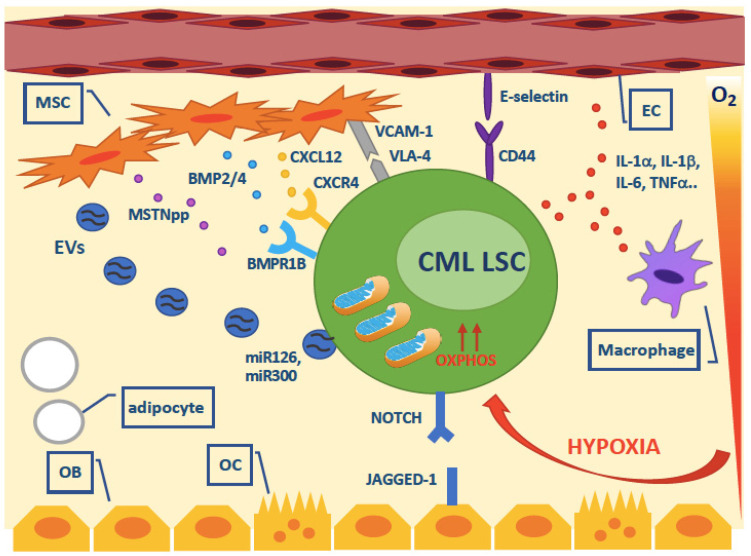
Overview of the cell extrinsic survival factors that have been implicated in CML LSC persistence. Abbreviations: MSC, mesenchymal stromal cell; EC, endothelial cell; OB, osteoblast; OC, osteoclast; EVs, extracellular vesicles.

An overview of the role of cell extrinsic factors from the BMM on LSC survival is presented in Figure 4.

## 6. Will We Ever Be Able to Kill CML LSCs?

To what extent our study models can faithfully recapitulate what happens in vivo in CML patients? Despite technical advances and deeper and deeper knowledge about the different ‘game players’, it is likely that the observations made in vitro be insufficient or inadequate to account for what happens in vivo in CML patients.

Are LSC and BMM features the same in all the patients? Most probably not. How many different ‘LSC’ do exist? How many ‘BMM’ do exist? Besides inter-patient variability, intra-patient variability must be taken into account, too. Recent single cell studies have demonstrated that LSCs in CML (and most probably in all leukemias) exhibit subclone-specific heterogeneity [125,126]. In other words, the LSC pool (in individual patients and overall) consists of heterogeneous subpopulations of cells with different molecular expression profiles and, likely, different patterns of cell surface antigens. Therefore, not only across different patients but also in the same patient, not all the LSCs may express the same targets. Small subclones lacking the target antigen would thus survive therapy. Moreover, given the multitude, and the redundancy, of survival pathways/mechanisms that LSCs may activate to sustain their persistence, targeting only one pathway or mechanism might be ineffective, or might be susceptible to the rapid development of resistance because of the activation of another, by-pass mechanism.

**Figure 5:**
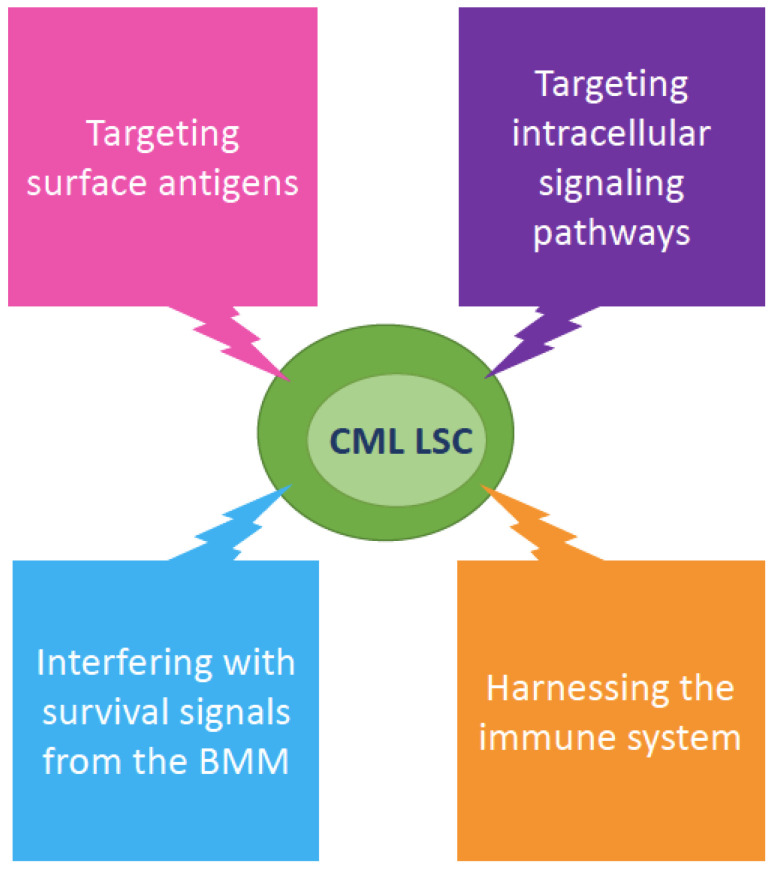
Overview of the therapeutic strategies that have been proposed to eradicate CML LSCs. Abbreviations: BMM, bone marrow microenvironment.

Another critical issue is represented by the actual feasibility of the various LSC-killing strategies that have so far been proposed. Several unique, LSC-specific pathways we might interfere with without affecting normal HSCs are now known. Similarly, several key interactions and regulators of LSC fate in the BMM have been identified and characterized (Figure 5).

However, despite the striking efficacy shown in vitro and most frequently also in mouse models by several approaches aimed to specifically target CML LSCs (recently reviewed by Yung et al. [127]), this has so far been translated into relatively few clinical trials (listed in Table 2). And most of these trials have, so far, exhibited relatively disappointing results, as discussed above.

Overall, a multitude of targets and candidate drugs/strategies have been proposed that would deserve clinical investigation. And several different patient settings can be envisioned for clinical trials evaluating treatment intensification with TKIs plus such drugs: (i) all newly diagnosed patients with a high LSC burden (e.g, in terms of CD93+ cells), or (ii) all patients aiming to TFR, or (iii) only those patients with residual disease above a certain threshold, or (iv) only those patients who have failed a previous TFR attempt. How long will it take before we will have demonstrated the safety and efficacy of one or more combinations and defined the best patient population where to apply them?

CML is not a fatal leukemia any longer. The great majority of newly diagnosed patients will achieve an optimal response and will most likely die from another cause [6] and this can be achieved with oral drugs that have, in most of the cases, limited and manageable side effects. Even patients who do not achieve an optimal response are often not in a condition of immediate risk. Survival curves clearly show that the great majority of patients who do not achieve DMR will fare well anyway [128,129,130].

If LSC eradication is meant to prevent resistance and progression, it can be objected that nowadays very few patients progress and face a dismal outcome. If LSC eradication is meant to enhance MRD clearance and/or the possibility to successfully discontinue TKI treatment, it can be objected that a combination therapy of TKIs plus an investigational agent with a less convenient schedule of administration and/or a less consolidated safety profile may not be worth the aim. These are probably the reasons why very few clinical trials have so far stemmed from the wealth of knowledge summarized above.

Last but not least—should we actually wish to go for the elimination of CML cells responsible for disease recurrence, are we really sure we know who our enemy is?

## 7. Do We Really Need to Kill CML LSCs?

Most patients who maintain TFR continue to harbor low levels of *BCR-ABL1*-positive cells. This has been demonstrated at various levels: (i) by using DNA-based PCR approaches, that have the drawback of being expensive and labor intensive (*BCR-ABL1* genomic breakpoints are patient-specific, thus they need to be characterized and individual PCR assays need to be set up and optimized to monitor each patient) but hold the advantage of picking *BCR-ABL1*-positive cells irrespective of their transcript expression levels [131]; (ii) by using more sensitive approaches for *BCR-ABL1* transcript detection, like digital PCR [132]; (iii) by using flow cytometry to enumerate the residual CD26+ cells [44]. All the studies were concordant in explaining their findings with the persistence of *BCR-ABL1*-positive LSCs. The lack of leukemia recurrence in these patients was then explained assuming a limited capacity for these residual cells to regenerate leukemia, that might be imputed: (i) to heterogeneity in the leukemogenic potential of *BCR-ABL1*-positive LSCs, and/or (ii) to restrictions imposed by specific microenvironmental and/or immune factors. Very recently, Pagani et al. [133] have provided a third explanation. They have investigated the lineage of residual CML cells in 20 patients in TFR for at least one year using an elegant approach of fluorescence-activated cell sorting followed by DNA PCR. *BCR-ABL1*-positive cells were thus searched in the granulocyte, monocyte, B cell, T cell, and NK cell compartments. Interestingly (and unexpectedly), residual *BCR-ABL1*-positive cells were identified predominantly in the lymphoid compartment and never in granulocytes. This means that *BCR-ABL1* positivity in PB of patients in TFR does not necessarily imply the persistence of residual multipotent LSCs.

A role for the immune control has indeed been suggested by several studies (recently reviewed by Hsieh et al. [134]). If residual LSCs persist and the immune system is the key to keep them in check, efforts towards sensitive LSC identification and quantitation and/or LSC phenotypical and functional characterization might be useless. Or, it might well be that a combination of factors (LSC-specific survival pathways + BMM protection + immune control) each to a variable extent, cooperate in these patients. If so, the complexity of the interplay among variables would hamper the possibility to predict which patients will truly need treatment intensification with LSC eradicating approaches.

## 8. Conclusions

Can we really improve upon TKI results in CML? And should we? Despite the widespread belief that the ultimate, future endpoint in CML has to be LSC eradication, the road is still long, rough and winding. Many wonder if this is worth the effort.

Nowadays, we can take advantage from very powerful and sophisticated technical capabilities, like single cell sequencing, multiomics, time lapse in vivo imaging etc that can offer unprecedented accuracy and resolution in the study of LSCs and their niches. Nevertheless, the full picture remains elusive. It is still challenging to recapitulate such a complex biology given that not all cell types in BM niches are known, that the peculiar and stringent physical and biochemical conditions of these niches (e.g., the low oxygen tension, the exposition to soluble factors..) can be reproduced only roughly, that cells before and after being placed in hypoxia for the duration of the experiment are collected and processed in normoxia, etc.

Anyway, CML has always been, and remains, an important model disease. CML LSCs have, probably, been more extensively studied that any other. The same applies to the influence of BMM on LSC survival. Many findings as well as techniques and experimental approaches set up and optimized in the CML setting may find further developments and applications in other disease models that are far behind. Thus, besides filling the textbooks, the two decades of research on CML LSC biology will find their clinical translation in many ways and in many fields.

## Figures and Tables

**Table 1:** Milestones in CML response to therapy.

Level	Abbreviation	Definition
Complete Hematological Response	CHR	Normalization of blood cell count
Complete Cytogenetic Response	CCyR	No metaphases positive for the Philadelphia chromosome out of 20 total metaphases examined by chromosome banding analysis
Major Molecular Response	MMR	3-log reduction in *BCR-ABL1* transcript levels (from a standardized baseline) as assessed by real time quantitative PCR
Deep Molecular Response	MR^4^	4-log reduction in *BCR-ABL1* transcript levels
	MR^4.5^	4.5-log reduction in *BCR-ABL1* transcript levels
	MR^5^	5-log reduction in *BCR-ABL1* transcript levels

**Table 2:** List of compounds that have been evaluated, in combination with TKIs, in clinical trials aimed to address LSC-targeting strategies in CML patients (data from clinicaltrials.gov).

Drug	Target	Trial ID	Status
LDE225 (sonidegib)	SMO	NCT01456676	Completed
PF-04449913 (glasdegib)	SMO	NCT00953758	Completed
BMS-833923	SMO	NCT01218477	Completed
RAD001	mTOR	NCT01188889	Withdrawn
LBH589 (panobinostat)	Histone deacetylases	NCT00686218 NCT00451035	Completed Completed
As_2_O_3_	PML	NCT01397734	Terminated
Chloroquine	Autophagy	NCT01227135	Completed
Zileuton	ALOX5	NCT01130688	Terminated
Ruxolitinib	JAK2	NCT01751425 NCT01702064 NCT02253277 NCT02973711 NCT03610971 NCT03654768	Terminated Completed Completed Withdrawn Recruiting Recruiting
PRI-274	CBP/β-catenin	NCT01606579	Completed
Pioglitazone	PPARγ/STAT5	NCT02730195 NCT04883125 NCT02852486 NCT02852486 NCT02889003 NCT02767063	Terminated Completed Active, not recruting Active, not recruiting Recruiting Recruiting

## References

[1] Soverini S., Mancini M., Bavaro L., Cavo M., Martinelli G. (2018). Chronic myeloid leukemia: The paradigm of targeting oncogenic tyrosine kinase signaling and counteracting resistance for successful cancer therapy. Mol. Cancer.

[2] Soverini S., Martinelli G., Iacobucci I., Baccarani M. (2008). Imatinib mesylate for the treatment of chronic myeloid leukemia. Expert Rev. Anticancer Ther..

[3] Soverini S., De Benedittis C., Mancini M., Martinelli G. (2016). Best practices in chronic myeloid leukemia monitoring and management. Oncologist.

[4] Bavaro L., Martelli M., Cavo M., Soverini S. (2019). Mechanisms of disease progression and resistance to tyrosine kinase inhibitor therapy in chronic myeloid leukemia: An update. Int. J. Mol. Sci..

[5] Hochhaus A., Baccarani M., Silver R.T., Schiffer C., Apperley J.F., Cervantes F., Clark R.E., Cortes J.E., Deininger M.W., Guilhot F. (2020). European LeukemiaNet 2020 recommendations for treating chronic myeloid leukemia. Leukemia.

[6] Bower H., Bjorkholm M., Dickman P.W., Hoglund M., Lambert P.C., Andersson T.M. (2016). Life expectancy of patients with chronic myeloid leukemia approaches the life expectancy of the general population. J. Clin. Oncol..

[7] Mahon F.X., Rea D., Guilhot J., Guilhot F., Huguet F., Nicolini F., Legros L., Charbonnier A., Guerci A., Varet B. (2010). Discontinuation of imatinib in patients with chronic myeloid leukaemia who have maintained complete molecular remission for at least 2 years: The prospective, multicentre Stop Imatinib (STIM) trial. Lancet Oncol..

[8] Soverini S., Rosti G., Baccarani M., Martinelli G. (2014). Molecular monitoring. Curr. Hematol. Malig. Rep..

[9] Ross D.M., Hughes T.P. (2020). Treatment-free remission in patients with chronic myeloid leukaemia. Nat. Rev. Clin. Oncol..

[10] Soverini S., Bassan R., Lion T. (2019). Treatment and monitoring of Philadelphia chromosome-positive leukemia patients: Recent advances and remaining challenges. J. Hematol. Oncol..

[11] Eisterer W., Jiang X., Christ O., Glimm H., Lee K.H., Pang E., Lambie K., Shaw G., Holyoake T.L., Petzer A.L. (2005). Different subsets of primary chronic myeloid leukemia stem cells engraft immunodeficient mice and produce a model of the human disease. Leukemia.

[12] Kinstrie R., Karamitros D., Goardon N., Morrison H., Hamblin M., Robinson L., Clark R.E., Copland M., Vyas P. (2016). Heterogeneous leukemia stem cells in myeloid blast phase chronic myeloid leukemia. Blood Adv..

[13] Zhang B., Li L., Ho Y., Li M., Marcucci G., Tong W., Bhatia R. (2016). Heterogeneity of leukemia-initiating capacity of chronic myelogenous leukemia stem cells. J. Clin. Investig..

[14] Bose S., Deininger M., Gora-Tybor J., Goldman J.M., Melo J.V. (1998). The presence of typical and atypical BCR-ABL fusion genes in leukocytes of normal individuals: Biologic significance and implications for the assessment of minimal residual disease. Blood.

[15] Biernaux C., Loos M., Sels A., Huez G., Stryckmans P. (1995). Detection of major bcr-abl gene expression at a very low level in blood cells of some healthy individuals. Blood.

[16] Huang W., Liu B., Eklund E.A. (2020). Investigating the role of the innate immune response in relapse or blast crisis in chronic myeloid leukemia. Leukemia.

[17] Holyoake T., Jiang X., Eaves C., Eaves A. (1999). Isolation of a highly quiescent subpopulation of primitive leukemic cells in chronic myeloid leukemia. Blood.

[18] Graham S.M., Jorgensen H.G., Allan E., Pearson C., Alcorn M.J., Richmond L., Holyoake T.L. (2002). Primitive, quiescent, Philadelphia-positive stem cells from patients with chronic myeloid leukemia are insensitive to STI571 in vitro. Blood.

[19] Holtz M.S., Slovak M.L., Zhang F., Sawyers C.L., Forman S.J., Bhatia R. (2002). Imatinib mesylate (STI571) inhibits growth of primitive malignant progenitors in chronic myelogenous leukemia through reversal of abnormally increased proliferation. Blood.

[20] Bhatia R., Holtz M., Niu N., Gray R., Snyder D.S., Sawyers C.L., Arber D.A., Slovak M.L., Forman S.J. (2003). Persistence of malignant hematopoietic progenitors in chronic myelogenous leukemia patients in complete cytogenetic remission following imatinib mesylate treatment. Blood.

[21] Chu S., McDonald T., Lin A., Chakraborty S., Huang Q., Snyder D.S., Bhatia R. (2011). Persistence of leukemia stem cells in chronic myelogenous leukemia patients in prolonged remission with imatinib treatment. Blood.

[22] Chomel J.C., Bonnet M.L., Sorel N., Bertrand A., Meunier M.C., Fichelson S., Melkus M., Bennaceur-Griscelli A., Guilhot F., Turhan A.G. (2011). Leukemic stem cell persistence in chronic myeloid leukemia patients with sustained undetectable molecular residual disease. Blood.

[23] Chomel J.C., Bonnet M.L., Sorel N., Sloma I., Bennaceur-Griscelli A., Rea D., Legros L., Marfaing-Koka A., Bourhis J.H., Ame S. (2016). Leukemic stem cell persistence in chronic myeloid leukemia patients in deep molecular response induced by tyrosine kinase inhibitors and the impact of therapy discontinuation. Oncotarget.

[24] Corbin A.S., Agarwal A., Loriaux M., Cortes J., Deininger M.W., Druker B.J. (2011). Human chronic myeloid leukemia stem cells are insensitive to imatinib despite inhibition of BCR-ABL activity. J. Clin. Investig..

[25] Hamilton A., Helgason G.V., Schemionek M., Zhang B., Myssina S., Allan E.K., Nicolini F.E., Muller-Tidow C., Bhatia R., Brunton V.G. (2012). Chronic myeloid leukemia stem cells are not dependent on Bcr-Abl kinase activity for their survival. Blood.

[26] Modi H., McDonald T., Chu S., Yee J.K., Forman S.J., Bhatia R. (2007). Role of BCR/ABL gene-expression levels in determining the phenotype and imatinib sensitivity of transformed human hematopoietic cells. Blood.

[27] Kumari A., Brendel C., Hochhaus A., Neubauer A., Burchert A. (2012). Low BCR-ABL expression levels in hematopoietic precursor cells enable persistence of chronic myeloid leukemia under imatinib. Blood.

[28] Schofield R. (1978). The relationship between the spleen colony-forming cell and the haemopoietic stem cell. Blood Cells.

[29] Janssen J.J., Deenik W., Smolders K.G., van Kuijk B.J., Pouwels W., Kelder A., Cornelissen J.J., Schuurhuis G.J., Ossenkoppele G.J. (2012). Residual normal stem cells can be detected in newly diagnosed chronic myeloid leukemia patients by a new flow cytometric approach and predict for optimal response to imatinib. Leukemia.

[30] Mustjoki S., Rohon P., Rapakko K., Jalkanen S., Koskenvesa P., Lundan T., Porkka K. (2010). Low or undetectable numbers of Philadelphia chromosome-positive leukemic stem cells (Ph(+)CD34(+)CD38(neg)) in chronic myeloid leukemia patients in complete cytogenetic remission after tyrosine kinase inhibitor therapy. Leukemia.

[31] Mustjoki S., Richter J., Barbany G., Ehrencrona H., Fioretos T., Gedde-Dahl T., Gjertsen B.T., Hovland R., Hernesniemi S., Josefsen D. (2013). Impact of malignant stem cell burden on therapy outcome in newly diagnosed chronic myeloid leukemia patients. Leukemia.

[32] Baccarani M., Deininger M.W., Rosti G., Hochhaus A., Soverini S., Apperley J.F., Cervantes F., Clark R.E., Cortes J.E., Guilhot F. (2013). European LeukemiaNet recommendations for the management of chronic myeloid leukemia: 2013. Blood.

[33] Thielen N., Richter J., Baldauf M., Barbany G., Fioretos T., Giles F., Gjertsen B.T., Hochhaus A., Schuurhuis G.J., Sopper S. (2016). Leukemic stem cell quantification in newly diagnosed patients with chronic myeloid leukemia predicts response to nilotinib therapy. Clin. Cancer Res..

[34] Valent P., Sadovnik I., Eisenwort G., Bauer K., Herrmann H., Gleixner K.V., Schulenburg A., Rabitsch W., Sperr W.R., Wolf D. (2019). Immunotherapy-based targeting and elimination of leukemic stem cells in AML and CML. Int. J. Mol. Sci..

[35] Jaras M., Johnels P., Hansen N., Agerstam H., Tsapogas P., Rissler M., Lassen C., Olofsson T., Bjerrum O.W., Richter J. (2010). Isolation and killing of candidate chronic myeloid leukemia stem cells by antibody targeting of IL-1 receptor accessory protein. Proc. Natl. Acad. Sci. USA.

[36] Landberg N., Hansen N., Askmyr M., Agerstam H., Lassen C., Rissler M., Hjorth-Hansen H., Mustjoki S., Jaras M., Richter J. (2016). IL1RAP expression as a measure of leukemic stem cell burden at diagnosis of chronic myeloid leukemia predicts therapy outcome. Leukemia.

[37] Warda W., Larosa F., Neto Da Rocha M., Trad R., Deconinck E., Fajloun Z., Faure C., Caillot D., Moldovan M., Valmary-Degano S. (2019). CML hematopoietic stem cells expressing IL1RAP can be targeted by chimeric antigen receptor-engineered T cells. Cancer Res..

[38] Landberg N., von Palffy S., Askmyr M., Lilljebjorn H., Sanden C., Rissler M., Mustjoki S., Hjorth-Hansen H., Richter J., Agerstam H. (2018). CD36 defines primitive chronic myeloid leukemia cells less responsive to imatinib but vulnerable to antibody-based therapeutic targeting. Haematologica.

[39] Valent P., Sadovnik I., Racil Z., Herrmann H., Blatt K., Cerny-Reiterer S., Eisenwort G., Lion T., Holyoake T., Mayer J. (2014). DPPIV (CD26) as a novel stem cell marker in Ph+ chronic myeloid leukaemia. Eur. J. Clin. Investig..

[40] Herrmann H., Sadovnik I., Cerny-Reiterer S., Rulicke T., Stefanzl G., Willmann M., Hoermann G., Bilban M., Blatt K., Herndlhofer S. (2014). Dipeptidylpeptidase IV (CD26) defines leukemic stem cells (LSC) in chronic myeloid leukemia. Blood.

[41] Blatt K., Menzl I., Eisenwort G., Cerny-Reiterer S., Herrmann H., Herndlhofer S., Stefanzl G., Sadovnik I., Berger D., Keller A. (2018). Phenotyping and target expression profiling of CD34(+)/CD38(-) and CD34(+)/CD38(+) stem- and progenitor cells in acute lymphoblastic leukemia. Neoplasia.

[42] Culen M., Borsky M., Nemethova V., Razga F., Smejkal J., Jurcek T., Dvorakova D., Zackova D., Weinbergerova B., Semerad L. (2016). Quantitative assessment of the CD26+ leukemic stem cell compartment in chronic myeloid leukemia: Patient-subgroups, prognostic impact, and technical aspects. Oncotarget.

[43] Raspadori D., Pacelli P., Sicuranza A., Abruzzese E., Iurlo A., Cattaneo D., Gozzini A., Galimberti S., Barate C., Pregno P. (2019). Flow cytometry assessment of CD26(+) leukemic stem cells in peripheral blood: A simple and rapid new diagnostic tool for chronic myeloid leukemia. Cytom. B Clin. Cytom..

[44] Bocchia M., Sicuranza A., Abruzzese E., Iurlo A., Sirianni S., Gozzini A., Galimberti S., Aprile L., Martino B., Pregno P. (2018). Residual peripheral blood CD26(+) leukemic stem cells in chronic myeloid leukemia patients during TKI therapy and during treatment-free remission. Front. Oncol..

[45] Bocchia M., Sicuranza A., Pacelli P., Iurlo A., Abruzzese E., Galimberti S., Pregno P., Caocci G., Capodanno I., Crugnola M. (2020). Peripheral blood CD26+ leukemia stem cells monitoring in chronic myeloid leukemia patients from diagnosis to response to TKIS: Interim results of a multicenter prospective study (PROSPECTIVE FLOWERS). Blood.

[46] Willmann M., Sadovnik I., Eisenwort G., Entner M., Bernthaler T., Stefanzl G., Hadzijusufovic E., Berger D., Herrmann H., Hoermann G. (2018). Evaluation of cooperative antileukemic effects of nilotinib and vildagliptin in Ph(+) chronic myeloid leukemia. Exp. Hematol..

[47] Houshmand M., Garello F., Stefania R., Gaidano V., Cignetti A., Spinelli M., Fava C., Nikougoftar Zarif M., Galimberti S., Pungolino E. (2021). Targeting chronic myeloid leukemia stem/progenitor cells using venetoclax-loaded immunoliposome. Cancers.

[48] Goff D.J., Court Recart A., Sadarangani A., Chun H.J., Barrett C.L., Krajewska M., Leu H., Low-Marchelli J., Ma W., Shih A.Y. (2013). A Pan-BCL2 inhibitor renders bone-marrow-resident human leukemia stem cells sensitive to tyrosine kinase inhibition. Cell Stem Cell.

[49] Carter B.Z., Mak P.Y., Mu H., Zhou H., Mak D.H., Schober W., Leverson J.D., Zhang B., Bhatia R., Huang X. (2016). Combined targeting of BCL-2 and BCR-ABL tyrosine kinase eradicates chronic myeloid leukemia stem cells. Sci. Transl. Med..

[50] Kobayashi C.I., Takubo K., Kobayashi H., Nakamura-Ishizu A., Honda H., Kataoka K., Kumano K., Akiyama H., Sudo T., Kurokawa M. (2014). The IL-2/CD25 axis maintains distinct subsets of chronic myeloid leukemia-initiating cells. Blood.

[51] Sadovnik I., Hoelbl-Kovacic A., Herrmann H., Eisenwort G., Cerny-Reiterer S., Warsch W., Hoermann G., Greiner G., Blatt K., Peter B. (2016). Identification of CD25 as STAT5-dependent growth regulator of leukemic stem cells in Ph+ CML. Clin. Cancer Res..

[52] Sadovnik I., Herrmann H., Eisenwort G., Blatt K., Hoermann G., Mueller N., Sperr W.R., Valent P. (2017). Expression of CD25 on leukemic stem cells in BCR-ABL1(+) CML: Potential diagnostic value and functional implications. Exp. Hematol..

[53] Sadovnik I., Herrmann H., Blatt K., Eisenwort G., Mueller N., Stefanzl G., Hoermann G., Herndlhofer S., Bauer K., Peter B. (2016). Evaluation of cell surface markers and targets in leukemic stem cells (LSC) reveals distinct expression profiles, unique drug effects, and specific checkpoint regulation in AML LSC and CML LSC. Blood.

[54] Kinstrie R., Horne G.A., Morrison H., Moka H.A., Cassels J., Dunn K., Herzyk P., Irvine D.A., Copland M. (2015). CD93 is a novel biomarker of leukemia stem cells in chronic myeloid leukemia. Blood.

[55] Kinstrie R., Horne G.A., Morrison H., Irvine D., Munje C., Castaneda E.G., Moka H.A., Dunn K., Cassels J.E., Parry N. (2020). CD93 is expressed on chronic myeloid leukemia stem cells and identifies a quiescent population which persists after tyrosine kinase inhibitor therapy. Leukemia.

[56] Dierks C., Beigi R., Guo G.R., Zirlik K., Stegert M.R., Manley P., Trussell C., Schmitt-Graeff A., Landwerlin K., Veelken H. (2008). Expansion of Bcr-Abl-positive leukemic stem cells is dependent on Hedgehog pathway activation. Cancer Cell.

[57] Zhao C., Chen A., Jamieson C.H., Fereshteh M., Abrahamsson A., Blum J., Kwon H.Y., Kim J., Chute J.P., Rizzieri D. (2009). Hedgehog signalling is essential for maintenance of cancer stem cells in myeloid leukaemia. Nature.

[58] Reya T., Duncan A.W., Ailles L., Domen J., Scherer D.C., Willert K., Hintz L., Nusse R., Weissman I.L. (2003). A role for Wnt signalling in self-renewal of haematopoietic stem cells. Nature.

[59] Zhao C., Blum J., Chen A., Kwon H.Y., Jung S.H., Cook J.M., Lagoo A., Reya T. (2007). Loss of beta-catenin impairs the renewal of normal and CML stem cells in vivo. Cancer Cell.

[60] Riether C., Schurch C.M., Flury C., Hinterbrandner M., Druck L., Huguenin A.L., Baerlocher G.M., Radpour R., Ochsenbein A.F. (2015). Tyrosine kinase inhibitor-induced CD70 expression mediates drug resistance in leukemia stem cells by activating Wnt signaling. Sci. Transl. Med..

[61] Schurch C., Riether C., Matter M.S., Tzankov A., Ochsenbein A.F. (2012). CD27 signaling on chronic myelogenous leukemia stem cells activates Wnt target genes and promotes disease progression. J. Clin. Investig..

[62] Gregory M.A., Phang T.L., Neviani P., Alvarez-Calderon F., Eide C.A., O’Hare T., Zaberezhnyy V., Williams R.T., Druker B.J., Perrotti D. (2010). Wnt/Ca2+/NFAT signaling maintains survival of Ph+ leukemia cells upon inhibition of Bcr-Abl. Cancer Cell.

[63] Naka K., Hoshii T., Muraguchi T., Tadokoro Y., Ooshio T., Kondo Y., Nakao S., Motoyama N., Hirao A. (2010). TGF-beta-FOXO signalling maintains leukaemia-initiating cells in chronic myeloid leukaemia. Nature.

[64] Neviani P., Harb J.G., Oaks J.J., Santhanam R., Walker C.J., Ellis J.J., Ferenchak G., Dorrance A.M., Paisie C.A., Eiring A.M. (2013). PP2A-activating drugs selectively eradicate TKI-resistant chronic myeloid leukemic stem cells. J. Clin. Investig..

[65] Chen M., Gallipoli P., DeGeer D., Sloma I., Forrest D.L., Chan M., Lai D., Jorgensen H., Ringrose A., Wang H.M. (2013). Targeting primitive chronic myeloid leukemia cells by effective inhibition of a new AHI-1-BCR-ABL-JAK2 complex. J. Natl. Cancer Inst..

[66] Traer E., MacKenzie R., Snead J., Agarwal A., Eiring A.M., O’Hare T., Druker B.J., Deininger M.W. (2012). Blockade of JAK2-mediated extrinsic survival signals restores sensitivity of CML cells to ABL inhibitors. Leukemia.

[67] Eiring A.M., Page B.D.G., Kraft I.L., Mason C.C., Vellore N.A., Resetca D., Zabriskie M.S., Zhang T.Y., Khorashad J.S., Engar A.J. (2015). Combined STAT3 and BCR-ABL1 inhibition induces synthetic lethality in therapy-resistant chronic myeloid leukemia. Leukemia.

[68] Kuepper M.K., Butow M., Herrmann O., Ziemons J., Chatain N., Maurer A., Kirschner M., Maie T., Costa I.G., Eschweiler J. (2019). Stem cell persistence in CML is mediated by extrinsically activated JAK1-STAT3 signaling. Leukemia.

[69] Salomoni P., Pandolfi P.P. (2002). The role of PML in tumor suppression. Cell.

[70] Bernardi R., Guernah I., Jin D., Grisendi S., Alimonti A., Teruya-Feldstein J., Cordon-Cardo C., Simon M.C., Rafii S., Pandolfi P.P. (2006). PML inhibits HIF-1alpha translation and neoangiogenesis through repression of mTOR. Nature.

[71] Ito K., Bernardi R., Morotti A., Matsuoka S., Saglio G., Ikeda Y., Rosenblatt J., Avigan D.E., Teruya-Feldstein J., Pandolfi P.P. (2008). PML targeting eradicates quiescent leukaemia-initiating cells. Nature.

[72] Park C.S., Lewis A.H., Chen T.J., Bridges C.S., Shen Y., Suppipat K., Puppi M., Tomolonis J.A., Pang P.D., Mistretta T.A. (2019). A KLF4-DYRK2-mediated pathway regulating self-renewal in CML stem cells. Blood.

[73] Abraham S.A., Hopcroft L.E., Carrick E., Drotar M.E., Dunn K., Williamson A.J., Korfi K., Baquero P., Park L.E., Scott M.T. (2016). Dual targeting of p53 and c-MYC selectively eliminates leukaemic stem cells. Nature.

[74] Salati S., Salvestrini V., Carretta C., Genovese E., Rontauroli S., Zini R., Rossi C., Ruberti S., Bianchi E., Barbieri G. (2017). Deregulated expression of miR-29a-3p, miR-494-3p and miR-660-5p affects sensitivity to tyrosine kinase inhibitors in CML leukemic stem cells. Oncotarget.

[75] Pellicano F., Park L., Hopcroft L.E.M., Shah M.M., Jackson L., Scott M.T., Clarke C.J., Sinclair A., Abraham S.A., Hair A. (2018). hsa-mir183/EGR1-mediated regulation of E2F1 is required for CML stem/progenitor cell survival. Blood.

[76] Ruiz M.S., Sanchez M.B., Bonecker S., Furtado C., Koile D., Yankilevich P., Cranco S., Custidiano M.D.R., Freitas J., Moiraghi B. (2020). miRNome profiling of LSC-enriched CD34(+)CD38(-)CD26(+) fraction in Ph(+) CML-CP samples from Argentinean patients: A potential new pharmacogenomic tool. Front. Pharm..

[77] Zipeto M.A., Court A.C., Sadarangani A., Delos Santos N.P., Balaian L., Chun H.J., Pineda G., Morris S.R., Mason C.N., Geron I. (2016). ADAR1 activation drives leukemia stem cell self-renewal by impairing let-7 biogenesis. Cell Stem Cell.

[78] Wang W.Z., Pu Q.H., Lin X.H., Liu M.Y., Wu L.R., Wu Q.Q., Chen Y.H., Liao F.F., Zhu J.Y., Jin X.B. (2015). Silencing of miR-21 sensitizes CML CD34+ stem/progenitor cells to imatinib-induced apoptosis by blocking PI3K/AKT pathway. Leuk. Res..

[79] Mortensen M., Soilleux E.J., Djordjevic G., Tripp R., Lutteropp M., Sadighi-Akha E., Stranks A.J., Glanville J., Knight S., Jacobsen S.E. (2011). The autophagy protein Atg7 is essential for hematopoietic stem cell maintenance. J. Exp. Med..

[80] Rubinsztein D.C., Codogno P., Levine B. (2012). Autophagy modulation as a potential therapeutic target for diverse diseases. Nat. Rev. Drug Discov..

[81] Bellodi C., Lidonnici M.R., Hamilton A., Helgason G.V., Soliera A.R., Ronchetti M., Galavotti S., Young K.W., Selmi T., Yacobi R. (2009). Targeting autophagy potentiates tyrosine kinase inhibitor-induced cell death in Philadelphia chromosome-positive cells, including primary CML stem cells. J. Clin. Investig..

[82] Scott M.T., Korfi K., Saffrey P., Hopcroft L.E., Kinstrie R., Pellicano F., Guenther C., Gallipoli P., Cruz M., Dunn K. (2016). Epigenetic reprogramming sensitizes CML stem cells to combined EZH2 and tyrosine kinase inhibition. Cancer Discov..

[83] Zhang B., Strauss A.C., Chu S., Li M., Ho Y., Shiang K.D., Snyder D.S., Huettner C.S., Shultz L., Holyoake T. (2010). Effective targeting of quiescent chronic myelogenous leukemia stem cells by histone deacetylase inhibitors in combination with imatinib mesylate. Cancer Cell.

[84] Li L., Wang L., Li L., Wang Z., Ho Y., McDonald T., Holyoake T.L., Chen W., Bhatia R. (2012). Activation of p53 by SIRT1 inhibition enhances elimination of CML leukemia stem cells in combination with imatinib. Cancer Cell.

[85] Yuan H., Wang Z., Li L., Zhang H., Modi H., Horne D., Bhatia R., Chen W. (2012). Activation of stress response gene SIRT1 by BCR-ABL promotes leukemogenesis. Blood.

[86] Abraham A., Qiu S., Chacko B.K., Li H., Paterson A., He J., Agarwal P., Shah M., Welner R., Darley-Usmar V.M. (2019). SIRT1 regulates metabolism and leukemogenic potential in CML stem cells. J. Clin. Investig..

[87] Kuntz E.M., Baquero P., Michie A.M., Dunn K., Tardito S., Holyoake T.L., Helgason G.V., Gottlieb E. (2017). Targeting mitochondrial oxidative phosphorylation eradicates therapy-resistant chronic myeloid leukemia stem cells. Nat. Med..

[88] Yang K., Wang F., Zhang H., Wang X., Chen L., Su X., Wu X., Han Q., Chen Z., Chen Z.S. (2020). Target inhibition of CBP induced cell senescence in BCR-ABL- T315I mutant chronic myeloid leukemia. Front. Oncol..

[89] Chen Y., Hu Y., Zhang H., Peng C., Li S. (2009). Loss of the Alox5 gene impairs leukemia stem cells and prevents chronic myeloid leukemia. Nat. Genet..

[90] Chen Y., Peng C., Abraham S.A., Shan Y., Guo Z., Desouza N., Cheloni G., Li D., Holyoake T.L., Li S. (2014). Arachidonate 15-lipoxygenase is required for chronic myeloid leukemia stem cell survival. J. Clin. Investig..

[91] Gallipoli P., Pellicano F., Morrison H., Laidlaw K., Allan E.K., Bhatia R., Copland M., Jorgensen H.G., Holyoake T.L. (2013). Autocrine TNF-alpha production supports CML stem and progenitor cell survival and enhances their proliferation. Blood.

[92] Grockowiak E., Laperrousaz B., Jeanpierre S., Voeltzel T., Guyot B., Gobert S., Nicolini F.E., Maguer-Satta V. (2017). Immature CML cells implement a BMP autocrine loop to escape TKI treatment. Blood.

[93] Himburg H.A., Roos M., Fang T., Zhang Y., Termini C.M., Schlussel L., Kim M., Pang A., Kan J., Zhao L. (2020). Chronic myeloid leukemia stem cells require cell-autonomous pleiotrophin signaling. J. Clin. Investig..

[94] Muselli F., Peyron J.F., Mary D. (2019). Druggable biochemical pathways and potential therapeutic alternatives to target leukemic stem cells and eliminate the residual disease in chronic myeloid leukemia. Int. J. Mol. Sci..

[95] Sweet K., Hazlehurst L., Sahakian E., Powers J., Nodzon L., Kayali F., Hyland K., Nelson A., Pinilla-Ibarz J. (2018). A phase I clinical trial of ruxolitinib in combination with nilotinib in chronic myeloid leukemia patients with molecular evidence of disease. Leuk. Res..

[96] Irvine D.A., Zhang B., Kinstrie R., Tarafdar A., Morrison H., Campbell V.L., Moka H.A., Ho Y., Nixon C., Manley P.W. (2016). Deregulated hedgehog pathway signaling is inhibited by the smoothened antagonist LDE225 (Sonidegib) in chronic phase chronic myeloid leukaemia. Sci. Rep..

[97] Horne G.A., Stobo J., Kelly C., Mukhopadhyay A., Latif A.L., Dixon-Hughes J., McMahon L., Cony-Makhoul P., Byrne J., Smith G. (2020). A randomised phase II trial of hydroxychloroquine and imatinib versus imatinib alone for patients with chronic myeloid leukaemia in major cytogenetic response with residual disease. Leukemia.

[98] Baquero P., Dawson A., Mukhopadhyay A., Kuntz E.M., Mitchell R., Olivares O., Ianniciello A., Scott M.T., Dunn K., Nicastri M.C. (2019). Targeting quiescent leukemic stem cells using second generation autophagy inhibitors. Leukemia.

[99] Zhao Y., Masiello D., McMillian M., Nguyen C., Wu Y., Melendez E., Smbatyan G., Kida A., He Y., Teo J.L. (2016). CBP/catenin antagonist safely eliminates drug-resistant leukemia-initiating cells. Oncogene.

[100] Rousselot P., Prost S., Guilhot J., Roy L., Etienne G., Legros L., Charbonnier A., Coiteux V., Cony-Makhoul P., Huguet F. (2017). Pioglitazone together with imatinib in chronic myeloid leukemia: A proof of concept study. Cancer.

[101] Pagnano K.B.B., Lopes A.B.P., Miranda E.C., Delamain M.T., Duarte G.O., Rodrigues B.R.V., Povoa V.M.O., Furlin G.C.P., Vianna J.C., da Silva M.A.S. (2020). Efficacy and safety of pioglitazone in a phase 1/2 imatinib discontinuation trial (EDI-PIO) in chronic myeloid leukemia with deep molecular response. Am. J. Hematol..

[102] Selleri C., Maciejewski J.P., Pane F., Luciano L., Raiola A.M., Mostarda I., Salvatore F., Rotoli B. (1998). Fas-mediated modulation of Bcr/Abl in chronic myelogenous leukemia results in differential effects on apoptosis. Blood.

[103] Scarpa E.S., Ninfali P. (2015). Phytochemicals as innovative therapeutic tools against cancer stem cells. Int. J. Mol. Sci..

[104] Liskova A., Kubatka P., Samec M., Zubor P., Mlyncek M., Bielik T., Samuel S.M., Zulli A., Kwon T.K., Busselberg D. (2019). Dietary phytochemicals targeting cancer stem cells. Molecules.

[105] Kantarjian H.M., O’Brien S., Cortes J. (2013). Homoharringtonine/omacetaxine mepesuccinate: The long and winding road to food and drug administration approval. Clin. Lymphoma Myeloma Leuk..

[106] Mak D.H., Schober W.D., Chen W., Konopleva M., Cortes J., Kantarjian H.M., Andreeff M., Carter B.Z. (2009). Triptolide induces cell death independent of cellular responses to imatinib in blast crisis chronic myelogenous leukemia cells including quiescent CD34+ primitive progenitor cells. Mol. Cancer Ther..

[107] Gu Y., Chen T., Meng Z., Gan Y., Xu X., Lou G., Li H., Gan X., Zhou H., Tang J. (2012). CaMKII gamma, a critical regulator of CML stem/progenitor cells, is a target of the natural product berbamine. Blood.

[108] Wu E.J., Goussetis D.J., Beauchamp E., Kosciuczuk E.M., Altman J.K., Eklund E.A., Platanias L.C. (2014). Resveratrol enhances the suppressive effects of arsenic trioxide on primitive leukemic progenitors. Cancer Biol. Ther..

[109] Du Y., Xia Y., Pan X., Chen Z., Wang A., Wang K., Li J., Zhang J. (2014). Fenretinide targets chronic myeloid leukemia stem/progenitor cells by regulation of redox signaling. Antioxid. Redox Signal..

[110] Simsek T., Kocabas F., Zheng J., Deberardinis R.J., Mahmoud A.I., Olson E.N., Schneider J.W., Zhang C.C., Sadek H.A. (2010). The distinct metabolic profile of hematopoietic stem cells reflects their location in a hypoxic niche. Cell Stem Cell.

[111] Suda T., Takubo K., Semenza G.L. (2011). Metabolic regulation of hematopoietic stem cells in the hypoxic niche. Cell Stem Cell.

[112] Zhang H., Li H., Xi H.S., Li S. (2012). HIF1alpha is required for survival maintenance of chronic myeloid leukemia stem cells. Blood.

[113] Zhang B., Ho Y.W., Huang Q., Maeda T., Lin A., Lee S.U., Hair A., Holyoake T.L., Huettner C., Bhatia R. (2012). Altered microenvironmental regulation of leukemic and normal stem cells in chronic myelogenous leukemia. Cancer Cell.

[114] Zhang B., Chu S., Agarwal P., Campbell V.L., Hopcroft L., Jorgensen H.G., Lin A., Gaal K., Holyoake T.L., Bhatia R. (2016). Inhibition of interleukin-1 signaling enhances elimination of tyrosine kinase inhibitor-treated CML stem cells. Blood.

[115] Agarwal P., Isringhausen S., Li H., Paterson A.J., He J., Gomariz A., Nagasawa T., Nombela-Arrieta C., Bhatia R. (2019). Mesenchymal niche-specific expression of Cxcl12 controls quiescence of treatment-resistant leukemia stem cells. Cell Stem Cell.

[116] Laperrousaz B., Jeanpierre S., Sagorny K., Voeltzel T., Ramas S., Kaniewski B., Ffrench M., Salesse S., Nicolini F.E., Maguer-Satta V. (2013). Primitive CML cell expansion relies on abnormal levels of BMPs provided by the niche and on BMPRIb overexpression. Blood.

[117] Von Palffy S., Landberg N., Sanden C., Zacharaki D., Shah M., Nakamichi N., Hansen N., Askmyr M., Lilljebjorn H., Rissler M. (2020). A high-content cytokine screen identifies myostatin propeptide as a positive regulator of primitive chronic myeloid leukemia cells. Haematologica.

[118] Zhang B., Li M., McDonald T., Holyoake T.L., Moon R.T., Campana D., Shultz L., Bhatia R. (2013). Microenvironmental protection of CML stem and progenitor cells from tyrosine kinase inhibitors through N-cadherin and Wnt-beta-catenin signaling. Blood.

[119] Zhang B., Nguyen L.X.T., Li L., Zhao D., Kumar B., Wu H., Lin A., Pellicano F., Hopcroft L., Su Y.L. (2018). Bone marrow niche trafficking of miR-126 controls the self-renewal of leukemia stem cells in chronic myelogenous leukemia. Nat. Med..

[120] Silvestri G., Trotta R., Stramucci L., Ellis J.J., Harb J.G., Neviani P., Wang S., Eisfeld A.K., Walker C.J., Zhang B. (2020). Persistence of drug-resistant leukemic stem cells and impaired NK cell immunity in CML patients depend on MIR300 antiproliferative and PP2A-activating functions. Blood Cancer Discov..

[121] Krause D.S., Lazarides K., Lewis J.B., von Andrian U.H., Van Etten R.A. (2014). Selectins and their ligands are required for homing and engraftment of BCR-ABL1+ leukemic stem cells in the bone marrow niche. Blood.

[122] Krause D.S., Lazarides K., von Andrian U.H., Van Etten R.A. (2006). Requirement for CD44 in homing and engraftment of BCR-ABL-expressing leukemic stem cells. Nat. Med..

[123] Godavarthy P.S., Kumar R., Herkt S.C., Pereira R.S., Hayduk N., Weissenberger E.S., Aggoune D., Manavski Y., Lucas T., Pan K.T. (2020). The vascular bone marrow niche influences outcome in chronic myeloid leukemia via the E-selectin—SCL/TAL1—CD44 axis. Haematologica.

[124] Bowers M., Zhang B., Ho Y., Agarwal P., Chen C.C., Bhatia R. (2015). Osteoblast ablation reduces normal long-term hematopoietic stem cell self-renewal but accelerates leukemia development. Blood.

[125] Giustacchini A., Thongjuea S., Barkas N., Woll P.S., Povinelli B.J., Booth C.A.G., Sopp P., Norfo R., Rodriguez-Meira A., Ashley N. (2017). Single-cell transcriptomics uncovers distinct molecular signatures of stem cells in chronic myeloid leukemia. Nat. Med..

[126] Warfvinge R., Geironson L., Sommarin M.N.E., Lang S., Karlsson C., Roschupkina T., Stenke L., Stentoft J., Olsson-Stromberg U., Hjorth-Hansen H. (2017). Single-cell molecular analysis defines therapy response and immunophenotype of stem cell subpopulations in CML. Blood.

[127] Yung Y., Lee E., Chu H.T., Yip P.K., Gill H. (2021). Targeting abnormal hematopoietic stem cells in chronic myeloid leukemia and philadelphia chromosome-negative classical myeloproliferative neoplasms. Int. J. Mol. Sci..

[128] Hehlmann R., Muller M.C., Lauseker M., Hanfstein B., Fabarius A., Schreiber A., Proetel U., Pletsch N., Pfirrmann M., Haferlach C. (2014). Deep molecular response is reached by the majority of patients treated with imatinib, predicts survival, and is achieved more quickly by optimized high-dose imatinib: Results from the randomized CML-study IV. J. Clin. Oncol..

[129] Falchi L., Kantarjian H.M., Wang X., Verma D., Quintas-Cardama A., O’Brien S., Jabbour E.J., Ravandi-Kashani F., Borthakur G., Garcia-Manero G. (2013). Significance of deeper molecular responses in patients with chronic myeloid leukemia in early chronic phase treated with tyrosine kinase inhibitors. Am. J. Hematol..

[130] Kantarjian H., Cortes J.E. (2014). Complete cytogenetic response, not deep molecular response, is associated with survival in chronic myeloid leukemia. J. Clin. Oncol..

[131] Ross D.M., Branford S., Seymour J.F., Schwarer A.P., Arthur C., Bartley P.A., Slader C., Field C., Dang P., Filshie R.J. (2010). Patients with chronic myeloid leukemia who maintain a complete molecular response after stopping imatinib treatment have evidence of persistent leukemia by DNA PCR. Leukemia.

[132] Bernardi S., Malagola M., Zanaglio C., Polverelli N., Dereli Eke E., D’Adda M., Farina M., Bucelli C., Scaffidi L., Toffoletti E. (2019). Digital PCR improves the quantitation of DMR and the selection of CML candidates to TKIs discontinuation. Cancer Med..

[133] Pagani I.S., Dang P., Saunders V.A., Grose R., Shanmuganathan N., Kok C.H., Carne L., Rwodzi Z., Watts S., McLean J. (2020). Lineage of measurable residual disease in patients with chronic myeloid leukemia in treatment-free remission. Leukemia.

[134] Hsieh Y.C., Kirschner K., Copland M. (2021). Improving outcomes in chronic myeloid leukemia through harnessing the immunological landscape. Leukemia.

